# Supplementary material for: CLK2 mediates IκBα-independent early termination of NF-κB activation by inducing cytoplasmic redistribution and degradation
Source: Nat Commun. 2024 May 9;15:3901. doi: 10.1038/s41467-024-48288-z (PMC11082251; doi:10.1038/s41467-024-48288-z)
Supplement: Supplementary file 1 — Supplementary Information [file 41467_2024_48288_MOESM1_ESM.pdf]

Supplementary Information

CLK2 mediates IκBα-independent early termination of NF-κB activation by inducing cytoplasmic redistribution and degradation

Shang-Ze Li[#, 1, 3], Qi-Peng Shu[#, 1], Hai-Meng Zhou[1], Yu-Ying Liu[1], Meng-Qi Fan[1], Xin-Yi Liang[1], Lin-Zhi Qi[3], Ya-Nan He[1], Xue-Yi Liu[1], Xue-Hua Du[1], Xi-Chen Huang[1], Yu-zhen Chen[2], Run-Lei Du[*, 1], Yue-xiu Liang[*, 2] and Xiao-Dong Zhang[*, 1, 2, 4]

[1]Hubei Key Laboratory of Cell Homeostasis, College of Life Sciences, Wuhan University, Wuhan, 430072, Hubei, China

[2]Key Laboratory of Research on Clinical Molecular Diagnosis for High Incidence Diseases in Western Guangxi of Guangxi Higher Education Institutions & Department of Gynecology, Affiliated Hospital of Youjiang Medical University for Nationalities

[3]School of Medicine, Chongqing University, Chongqing, 400044, China;

[4]National Health Commission Key Laboratory of Birth Defect Research and Prevention & MOE Key Lab of Rare Pediatric Diseases, Department of Cell Biology and Genetics, School of Basic Medical Sciences, School of Basic Medical Sciences, Hengyang Medical School, University of South China, Hengyang, China;

[#]These authors contributed equally to this work

[*]Correspondence to: Run-Lei Du, Email: runleidu@whu.edu.cn; Yue-xiu Liang, 105706981@qq.com; Xiao-Dong Zhang, Email: zhangxd@whu.edu.cn

Supplementary Figure 1

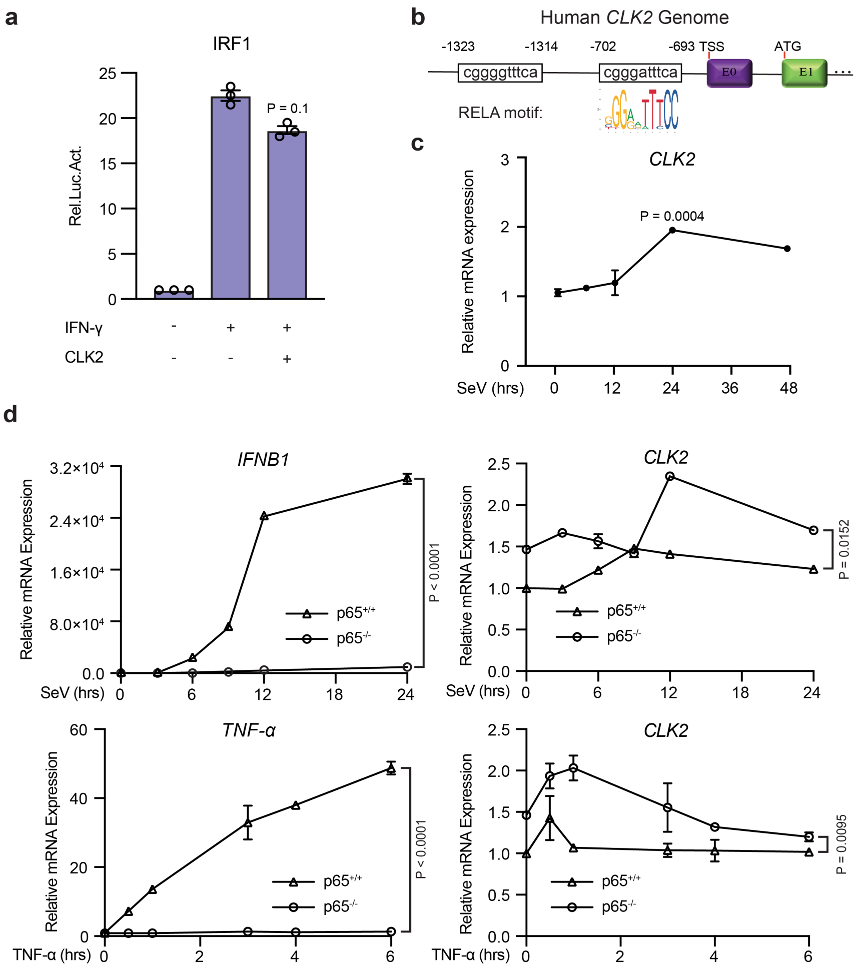

Supplementary Figure 1. CLK2 negatively regulates the transcriptional activation of NF-κB.

(**a**) IRF1 promoter activity after exposure to IFN-γ (100 ng/ml) for 12 hours in the presence or absence of CLK2 (800 ng) expression plasmids. (**b**) A schematic of the RELA motif in the human *CLK2* gene promoter. (**c**) The mRNA level of *CLK2* in HEK293T cells after SeV infection at the indicated time points was determined by real-time PCR analysis (n=3). (**d**) The mRNA levels of *IFNB1, TNF-α* and *CLK2* in $p65^{+/+}$ and $p65^{-/-}$ HEK293T cells after SeV or TNF-α infection at the indicated time points were determined by real-time PCR analysis (n=3). The data are representative of three independent experiments. The data are presented as the means ± SEMs (n=3 for **a**, **c** and **d**). Statistical significance was analyzed by two-tailed Student's *t* test (**a** and **c**) and two-tailed ANOVA (**d**) (p >0.05, not significant, *p < 0.05, **p < 0.01, ***p < 0.001, ****p < 0.0001). Source data (**a**-**d**) are provided as a Source Data file.

Supplementary Figure 2

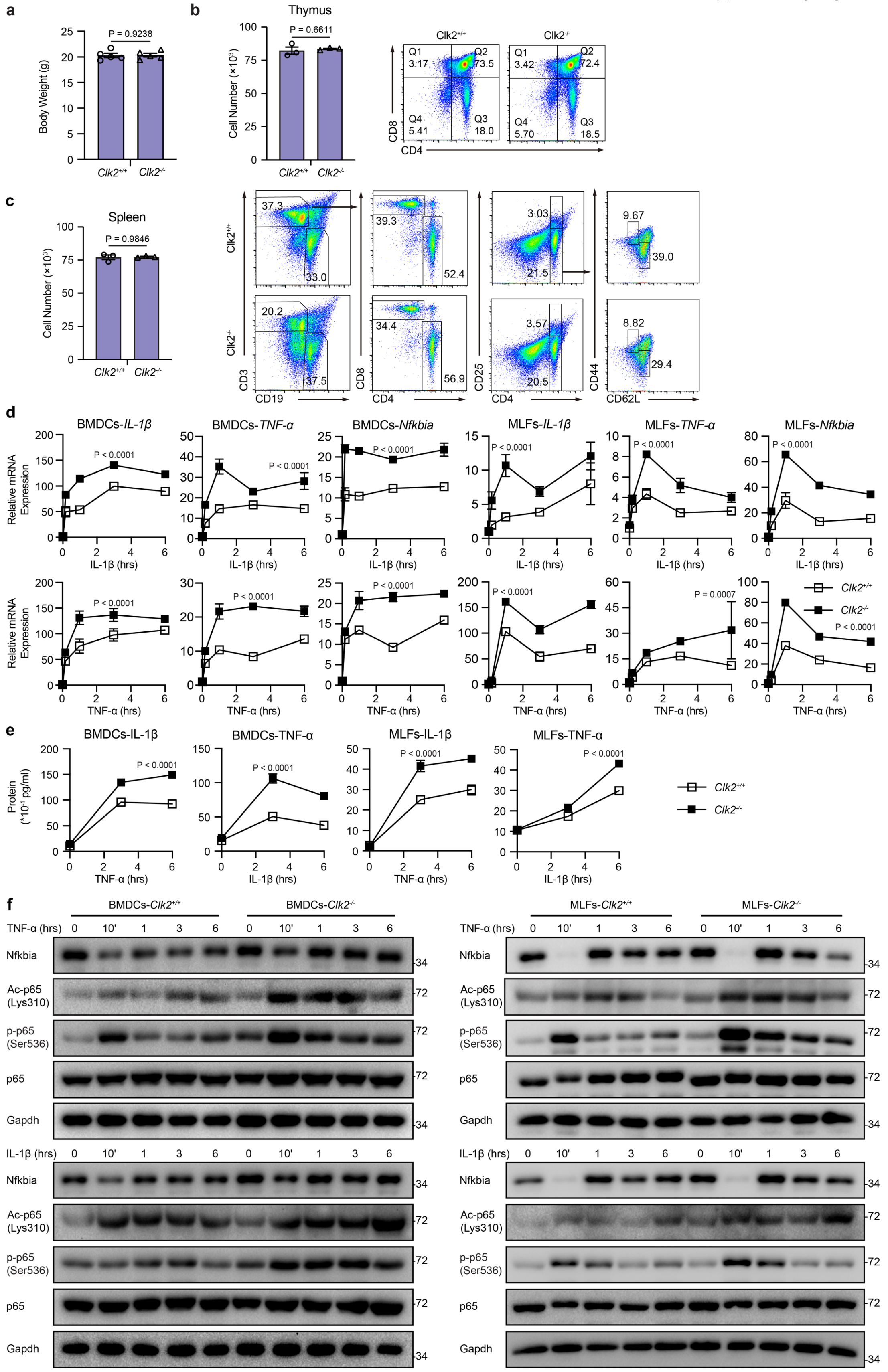

Supplementary Figure 2. Clk2 deficiency enhances the NF-κB-mediated inflammatory response.

(**a**) Body weights of *Clk2*$^{+/+}$ and *Clk2*$^{-/-}$ male mice at 7 weeks of age (n=5). (**b** and **c**) Flow cytometric analysis and quantitation of immune cells in the thymus (**b**) and spleen (**c**) of *Clk2*$^{+/+}$ and *Clk2*$^{-/-}$ male mice (n=3). (**d**) Real-time PCR analysis of *IL-1β*, *TNF-α* and *Nfkbia* mRNA levels in *Clk2*$^{+/+}$ and *Clk2*$^{-/-}$ BMDCs and MLFs stimulated with TNF-α (50 ng/ml) and IL-1β (50 ng/ml) for the indicated times (n=3). (**e**) ELISA analysis of IL-1β and TNF-α protein levels in *Clk2*$^{+/+}$ and *Clk2*$^{-/-}$ BMDCs and MLFs stimulated with TNF-α and IL-1β for the indicated times (n=3). (**f**) Western blot analysis of Nfkbia, Ac-p65^{K310}, p-p65^{S536}, p65 and Gapdh in *Clk2*$^{+/+}$ and *Clk2*$^{-/-}$ BMDCs and MLFs stimulated with TNF-α and IL-1β for the indicated times. The data are representative of three independent experiments. The data are presented as the means ± SEMs. Statistical significance was analyzed by two-tailed Student's *t* test (**a**-**c**) or two-tailed ANOVA (**d**, **e**) (p >0.05, not significant, *$p < 0.05$, **$p < 0.01$, ***$p < 0.001$, ****$p < 0.0001$). Source data (**a**-**f**) are provided as a Source Data file.

Supplementary Figure 3

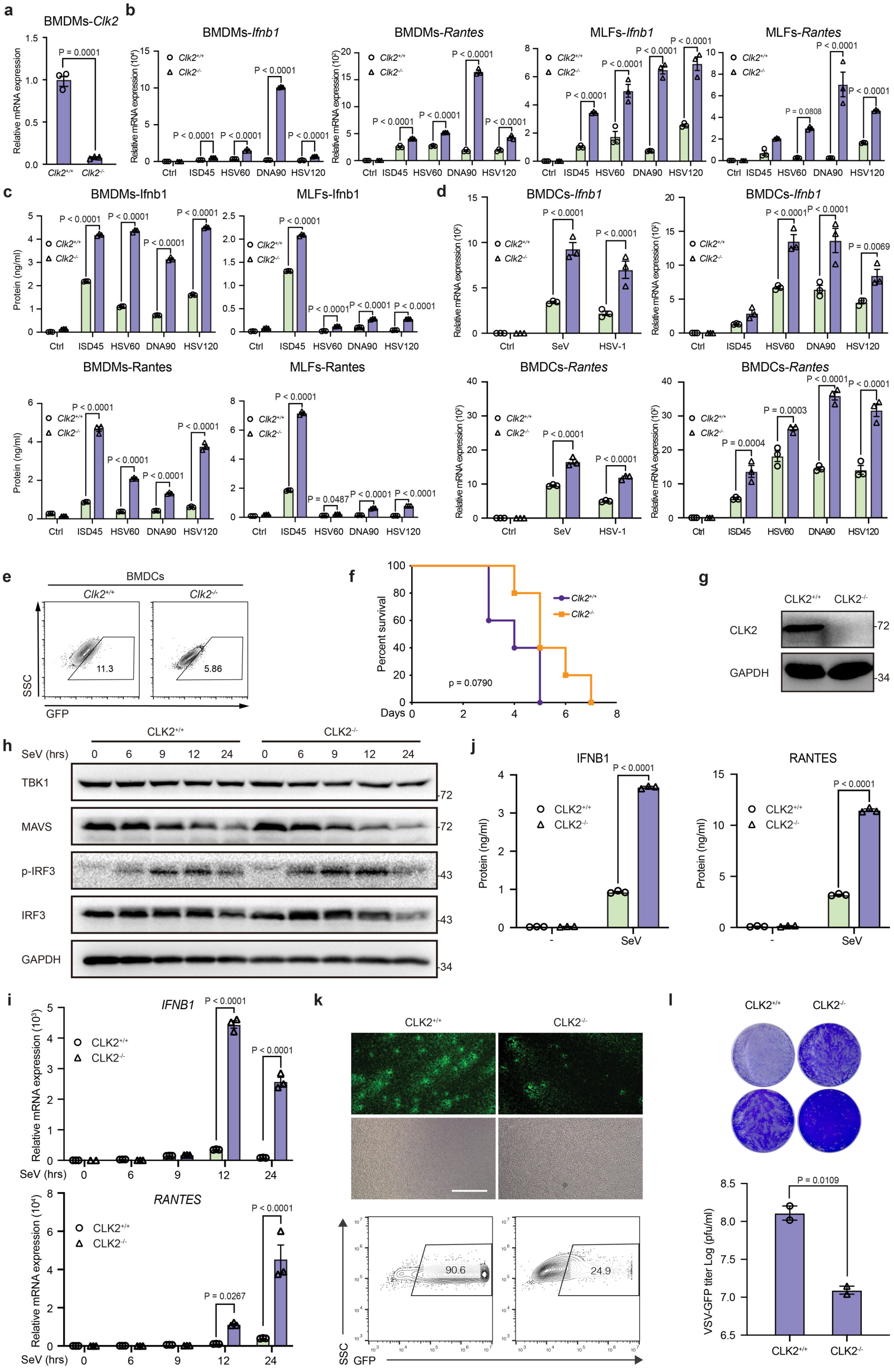

Supplementary Figure 3. CLK2 deficiency enhances virus-induced IFN-β production and the antiviral response. (**a**) Examination of Clk2-deficient BMDMs at the mRNA level (n=3). (**b**) Real-time PCR analysis of *Ifnb1* and *Rantes* mRNA levels in $Clk2^{+/+}$ and $Clk2^{-/-}$ BMDMs and MLFs transfected with ISD45, HSV60, DNA90, and HSV120 for 6 hours (n=3). (**c**) ELISA analysis of Ifnb1 and Rantes protein levels in $Clk2^{+/+}$ and $Clk2^{-/-}$ BMDMs and MLFs transfected with ISD45, HSV60, DNA90 and HSV120 for 6 hours (n=3). (**d**) Real-time PCR analysis of *Ifnb1* and *Rantes* mRNA levels in $Clk2^{+/+}$ and $Clk2^{-/-}$ BMDCs infected with SeV for 12 hours, infected with HSV-1 for 6 hours or transfected with ISD45, HSV60, DNA90 and HSV120 for 6 hours (n=3). (**e**) $Clk2^{+/+}$ and $Clk2^{-/-}$ BMDCs were infected with VSV-GFP at an MOI of 2 for 12 hours before flow cytometric analysis. (**f**) Survival analysis (Kaplan–Meier curves) of $Clk2^{+/+}$ and $Clk2^{-/-}$ mice at 6 weeks after infection with HSV-1 (2.4×10^6 pfu) via the tail vein and intraperitoneal injection. The survival of the mice was monitored for 8 days (n=5). (**g**) Western blot analysis of CLK2 protein levels in HEK293T cells to verify CLK2 deletion. (**h**) Western blot analysis of phosphorylated IRF3 and total TBK1, MAVS, IRF3 and GAPDH in $CLK2^{+/+}$ and $CLK2^{-/-}$ HEK293T cells infected with SeV at the indicated times. (**i**) Real-time PCR analysis of *IFNB1* and *RANTES* mRNA levels in $CLK2^{+/+}$ and $CLK2^{-/-}$ HEK293T cells infected with SeV for the indicated times (n=3). (**j**) ELISA analysis of IFNB1 and RANTES protein levels in the supernatants of $CLK2^{+/+}$ and $CLK2^{-/-}$ HEK293T cells infected with SeV for 24 hours (n=3). (**k**) The effects of CLK2 deficiency on VSV-GFP infection for 12 hours before phase contrast and fluorescence microscopy and flow cytometric analysis of $CLK2^{+/+}$ and $CLK2^{-/-}$ HEK293T cells. The scale bar is 500 µm. (**l**) $CLK2^{+/+}$ and $CLK2^{-/-}$ HEK293T cells were infected with VSV-GFP for 1 h, followed by culture for 24 hours with fresh medium. Then, the supernatants were subjected to plaque assays using Vero cells (n=2). The data are representative of three independent experiments. The data are presented as the means ± SEMs. Statistical significance was analyzed by two-tailed Student's *t* test (**a**, **l**) or two-tailed ANOVA (**b**-**d**, **i**-**j**) ($p > 0.05$, not significant, $^{*}p < 0.05$, $^{**}p < 0.01$, $^{***}p < 0.001$, $^{****}p < 0.0001$). Source data (**a**-**l**) are provided as a Source Data file.

Supplementary Figure 4

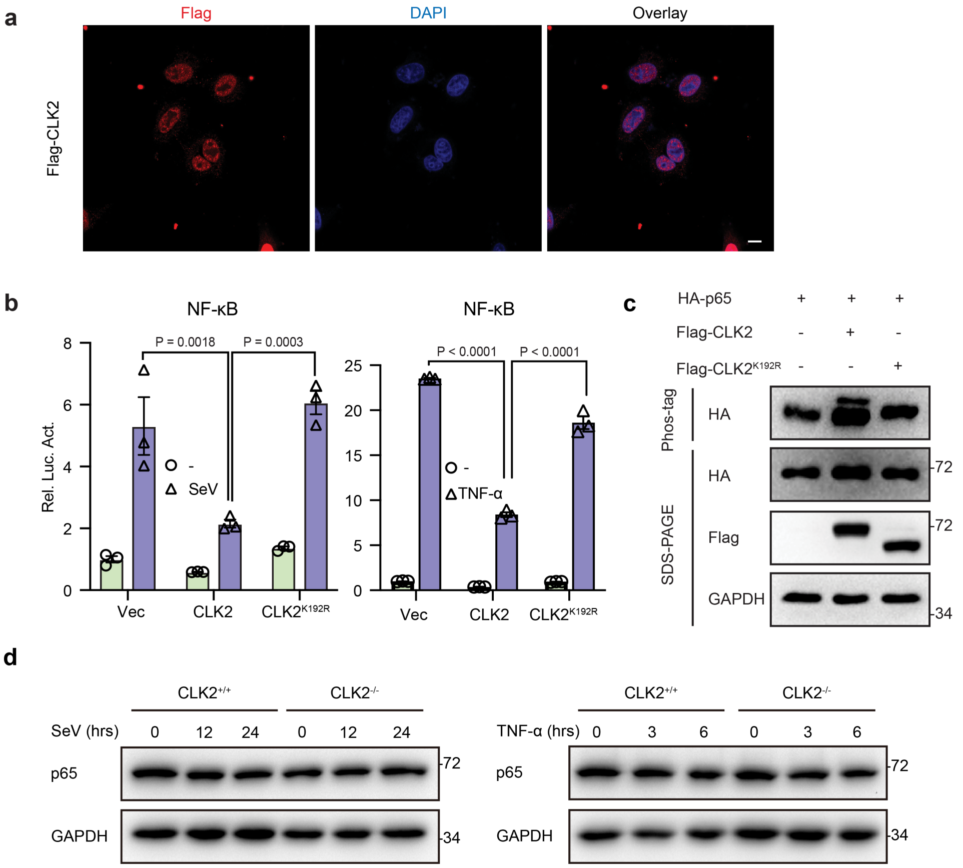

Supplementary Figure 4. CLK2 interacts with and phosphorylates p65 to reverse its nuclear localization.

(**a**) Immunofluorescence analysis of the localization of exogenous Flag-CLK2 in HEK293T cells. Images were obtained by fluorescence microscopy. The scale bar is 10 μm. (**b**) Luciferase reporter experiments analyzing NF-κB activity in HEK293T cells transfected with plasmids encoding CLK2 and $CLK2^{K192R}$ for 24 hours followed by stimulation with SeV or TNF-α for 12 hours (n=3). (**c**) Phos-tag SDS–PAGE analysis of the phosphorylation of p65 in the presence of CLK2 or $CLK2^{K192R}$ in HEK293T cells transfected with HA-p65 plus Flag-CLK2 or Flag-$CLK2^{K192R}$ before Phos-tag and SDS–PAGE were performed with anti-HA and anti-Flag antibodies. The upper shifted band represents the phosphorylated p65 protein. (**d**) Western blot analysis of p65 protein levels in $CLK2^{+/+}$ and $CLK2^{-/-}$ HEK293T cells infected with SeV or TNF-α at the indicated time points. The data are representative of three independent experiments. The data are presented as the means ± SEMs. Statistical significance was analyzed by two-tailed ANOVA (**b**) (p >0.05, not significant, *$p < 0.05$, **$p < 0.01$, ***$p < 0.001$, ****$p < 0.0001$). Source data (**b-d**) are provided as a Source Data file.

Supplementary Figure 5

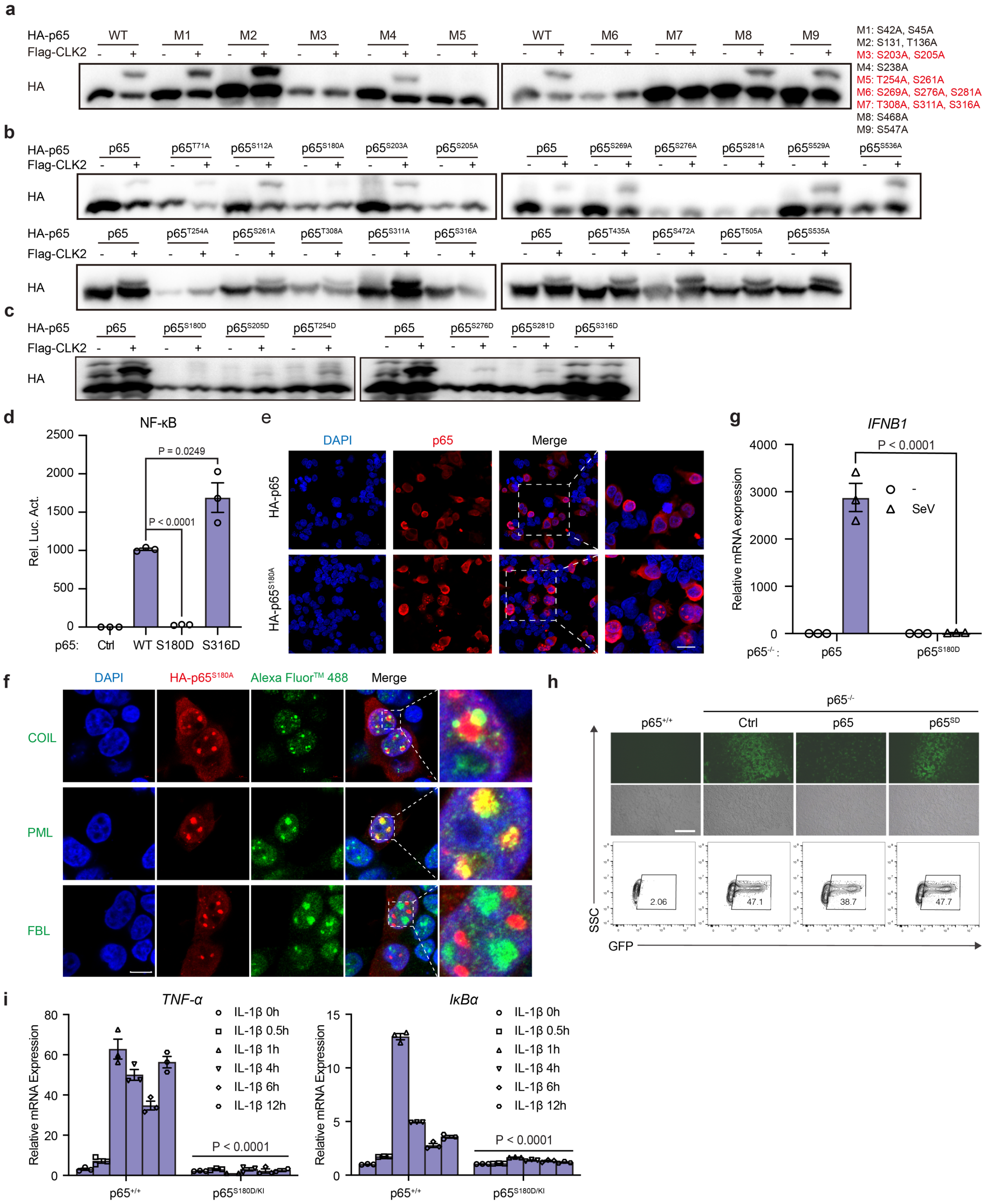

Supplementary Figure 5. The phosphorylation of p65 at Ser180 by CLK2 inhibits the transcriptional ability of activated NF-κB.

(**a**-**c**) Phos-tag SDS–PAGE analysis of the residues of p65 that are potentially phosphorylated by CLK2 using the indicated p65 mutants in HEK293T cells. The upper shifted band represents the phosphorylated p65 protein. (**d**) Luciferase reporter experiments analyzing NF-κB activity in HEK293T cells transfected with plasmids encoding p65, $p65^{S180D}$, and $p65^{S316D}$ for 24 hours (n=3). (**e**) Immunofluorescence analysis of exogenous HA-$p65^{S180A}$ and wild-type p65 localization in HEK293T cells. The scale bar is 40 μm. (**f**) The colocalization of HA-$p65^{S180A}$ with COIL, PML or FBL determined by immunofluorescence analysis in HEK293T cells. The scale bar is 40 μm. (**g**) Real-time PCR analysis of SeV-induced *IFNB1* mRNA expression in the presence of p65 and $p65^{S180D}$ in $p65^{-/-}$ HEK293T cells (n=3). (**h**) The indicated cells were infected with VSV-GFP at an MOI of 0.01 for 12 hours before phase contrast and fluorescence microscopy and flow cytometric analysis. The scale bar is 250 μm. (**i**) Real-time PCR analysis of *IκBα* and *TNF-α* mRNA levels in the indicated cells exposed to 20 ng/ml IL-1β for 0 h. 0.5, 1, 4, 6, and 12 hours (n=3). The data are representative of three independent experiments. The data are presented as the means ± SEMs. Statistical significance was analyzed by two-tailed Student's *t* test (**d**, **g**) or two-tailed ANOVA (**i**) (p >0.05, not significant, *$p < 0.05$, **$p < 0.01$, ***$p < 0.001$, ****$p < 0.0001$). Source data (**a**-**i**) are provided as a Source Data file.

Supplementary Figure 6

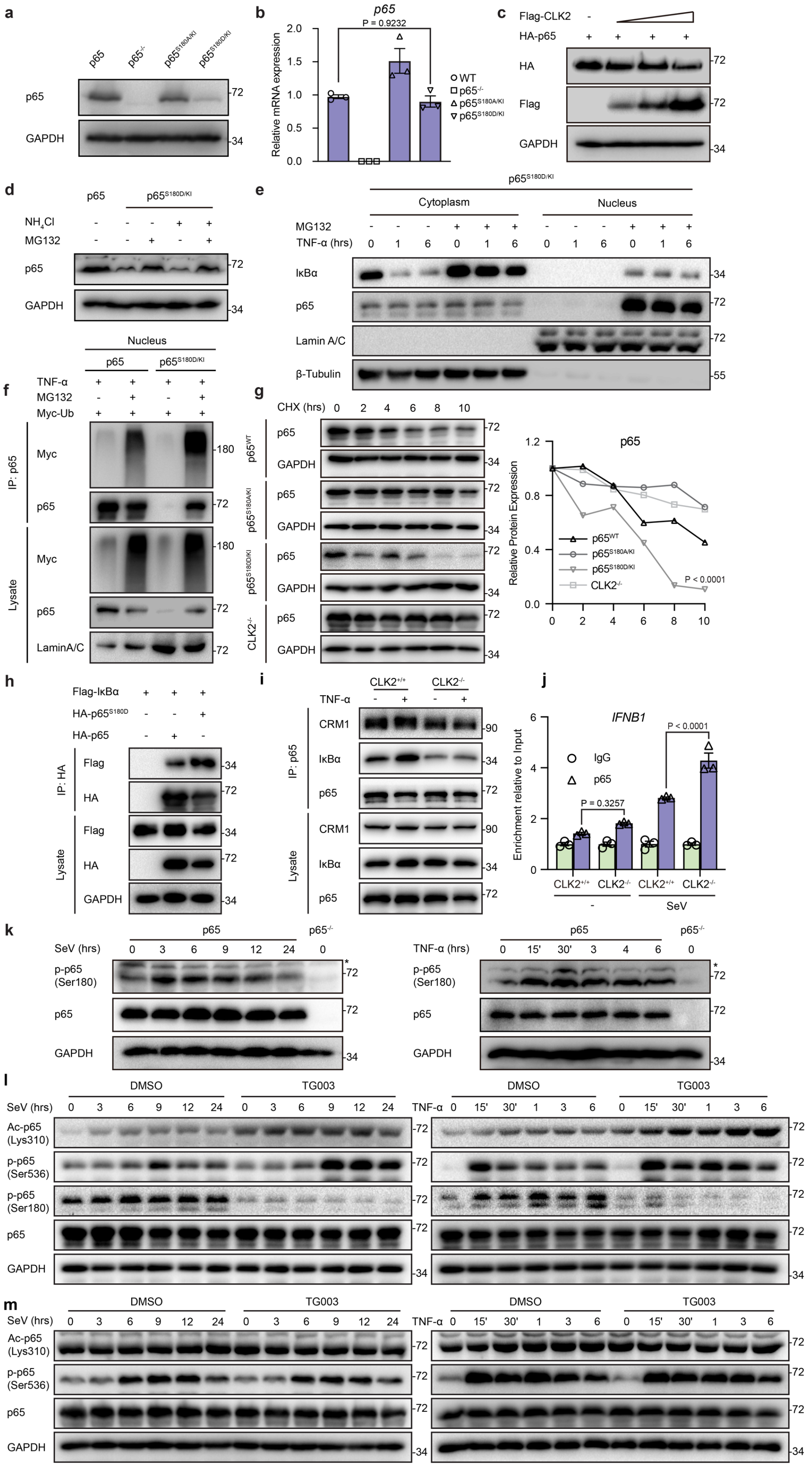

Supplementary Figure 6. Ser180 phosphorylation of p65 results in its degradation and nuclear export.

(**a**-**b**) Western blot and real-time PCR analysis of p65 protein and mRNA levels in $p65^{+/+}$, $p65^{-/-}$, $p65^{S180A/KI}$ and $p65^{S180D/KI}$ HEK293T cells. (**c**) Western blot analysis of p65 protein levels in the presence of increased CLK2 expression. (**d**) Western blot analysis of p65 protein levels in the presence of MG132 and NH_4Cl in $p65^{+/+}$ and $p65^{S180D/KI}$ HEK293T cells. (**e**) Cytoplasmic and nuclear fractions of $p65^{S180D/KI}$ HEK293T cells stimulated with TNF-α at the indicated time points with or without MG132 before immunoblot analysis with anti-p65, anti-IκBα, anti-Tubulin and anti-LaminA/C antibodies. (**f**) The nuclear fractions of $p65^{S180D/KI}$ and $p65^{+/+}$ HEK293T cells in the presence of TNF-α with or without MG132, followed by coimmunoprecipitation of p65 and Myc-Ub, and the proteins were analyzed by Western blotting. (**g**) Time course of cycloheximide (CHX) treatment of $p65^{+/+}$, $p65^{S180A}$, $p65^{S180D}$ and $CLK2^{-/-}$ HEK293T cells, as determined by Western blot analysis with anti-p65 and anti-GAPDH antibodies. The samples were derived from the same experiment and that blots were processes in parallel. (**h**) The interaction between $p65^{S180D}$ and IκBα in HEK293T cells transfected with HA-p65, HA-$p65^{S180D}$ or Flag-IκBα was determined by coimmunoprecipitation with the indicated antibodies and immunoblot analyses. (**i**) Coimmunoprecipitation of endogenous p65 and IκBα or CRM1 was performed in $CLK2^{+/+}$ and $CLK2^{-/-}$ HEK293T cells in the presence or absence of TNF-α stimulation. (**j**) ChIP assays were performed in $CLK2^{+/+}$ and $CLK2^{-/-}$ HEK293T cells stimulated with SeV, and the promotor of IFNB1 was examined by real-time qPCR (n=3). The sequences are presented in Supplementary Table 2. (**k**) Western blot analysis of phosphorylated Ser180 p65 and total p65 and GAPDH in $p65^{+/+}$ and $p65^{–/–}$ HEK293T cells stimulated with SeV and TNF-α at the indicated times. The upper band marked with asterisk indicates the unspecific band. (**l**) Western blot analysis of phosphorylated Ser180 and Ser536 p65, acetylated Lys310 p65, total p65 and GAPDH in HEK293T cells treated with or without TG003 and exposed to SeV or TNF-α for the indicated times. (**m**) Western blot analysis of phosphorylated Ser536 p65, acetylated Lys310 p65, total p65 and GAPDH in HEK293T-$CLK2^{-/-}$ cells treated with or without TG003 and exposed to SeV or TNF-α for the indicated times. The data are representative of three independent experiments. The data are presented as the means ± SEMs. Statistical significance was analyzed by two-tailed Student's *t* test (**b**) or two-tailed ANOVA (**g**, **j**) (p >0.05, not significant, *$p < 0.05$, **$p < 0.01$, ***$p < 0.001$, ****$p < 0.0001$). Source data (**a**-**l**) are provided as a Source Data file.

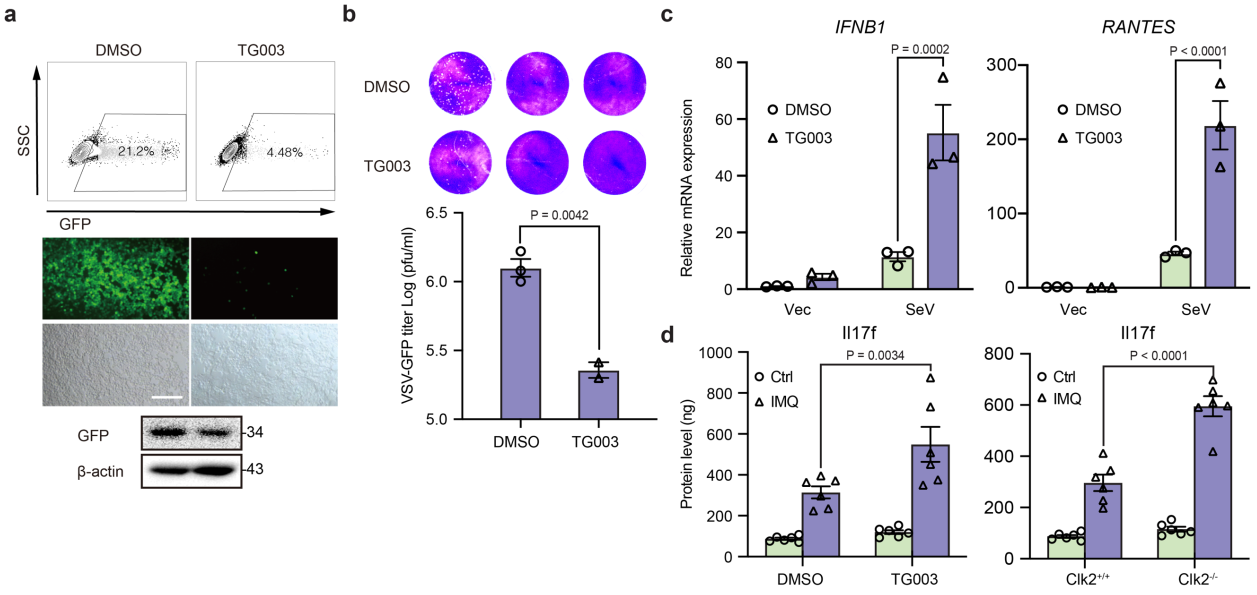

Supplementary Figure 7. The CLK2 inhibitor TG003 exerts effects *in vitro* and *in vivo*.

(**a**) HEK293T cells were infected with VSV-GFP at an MOI of 0.01 for 12 hours in the presence or absence of TG003 (20 μM) before phase contrast and fluorescence microscopy and flow cytometric analysis. The scale bar is 250 μm. (**b**) HEK293T cells were infected with VSV-GFP in the presence or absence of TG003 (20 μM) at an MOI of 0.01 for 1 hour in the presence or absence of TG003 (20 μM) before the medium was replaced, and the cells were cultured for 24 hours. Then, the supernatants were diluted and added to Vero cells for plaque assays (n=3). (**c**) Real-time PCR analysis of *IFNB1* and *RANTES* mRNA levels in the presence or absence of TG003 in HEK293T cells infected with SeV for 12 hours (n=3). (**d**) ELISA analysis of the Il-17f protein level after treatment of Clk2-deficient mice with IMQ for 6 days or after intraperitoneal injection of TG003. The data are representative of three independent experiments. The data are presented as the means ± SEMs. Statistical significance was analyzed by two-tailed Student's *t* test (**b**) or two-tailed ANOVA (**c**, **d**) (p >0.05, not significant, *$p < 0.05$, **$p < 0.01$, ***$p < 0.001$, ****$p < 0.0001$). Source data (**a-d**) are provided as a Source Data file.

Supplementary Table 1

Supplementary Table 1. The Antibody and Reagents

S.No.	Antibodies and Reagents	Source	Antibody dilution	Identifier
1	Mouse anti-human CLK2, Polyclonal	Sigma	1:1000 (WB)	Cat# HPA055366
2	Mouse anti-human CLK2, Polyclonal	Abclonal Technology	1:1000 (WB)	Cat# A7885
3	Rabbit anti-human IRF3, monoclonal	Cell Signaling Technology	1:1000 (WB)	Cat# 11904S
4	Rabbit anti-human p-IRF3 (Ser386), monoclonal	Cell Signaling Technology	1:1000 (WB)	Cat# 37829S
5	Mouse anti-human p65, monoclonal	Cell Signaling Technology	1:1000 (WB) 1:200 (IF) 1:100 (IP)	Cat# 6956S
6	Rabbit anti-human p-p65 (Ser536), monoclonal	Cell Signaling Technology	1:1000 (WB)	Cat# 3033S
7	Rabbit anti-human Ikbα, monoclonal	Cell Signaling Technology	1:1000 (WB)	Cat# 4812
8	Rabbit anti-human p-Ikbα (Ser32), monoclonal	Cell Signaling Technology	1:1000 (WB)	Cat# 2859S
9	Mouse anti-human β-Tubulin, Polyclonal	Cell Signaling Technology	1:1000 (WB)	Cat# 2146S
10	Rabbit anti-human β-actin, monoclonal	Abclonal Technology	1:50000 (WB)	Cat# AC026-200
11	Mouse anti-human GAPDH, monoclonal	Abclonal Technology	1:50000 (WB)	Cat# AC033
12	Mouse anti-human LaminA/C, monoclonal	Cell Signaling Technology	1:1000 (WB)	Cat# 4777S
13	Mouse anti-Flag, monoclonal	MBL	1:3000 (WB) 1:400 (IF) 1:150 (IP)	Cat# M185-3
14	Mouse anti-Myc, monoclonal	MBL	1:3000 (WB) 1:400 (IF) 1:150 (IP)	Cat# M192-3
15	Mouse anti-HA, monoclonal	MBL	1:3000 (WB) 1:400 (IF) 1:150 (IP)	Cat# M180-3
16	Rabbit anti-Flag, Polyclonal	MBL	1:3000 (WB) 1:400 (IF) 1:150 (IP)	Cat# PM020
17	Rabbit anti-Myc, Polyclonal	MBL	1:3000 (WB) 1:400 (IF) 1:150 (IP)	Cat# 562
18	Rabbit anti-HA, Polyclonal	MBL	1:3000 (WB) 1:400 (IF) 1:150 (IP)	Cat# 561
19	Rabbit IgG HRP Linked Antibody	Jackson ImmunoResearch	1:10000	Cat# 111-035-003
20	Mouse IgG HRP Linked Antibody	Jackson ImmunoResearch	1:10000	Cat# 115-035-003
21	Rabbit anti-human NF-κB1 p105/p50 (D4P4D), monoclonal	Cell Signaling Technology	1:1000 (WB)	Cat# 13586
22	Rabbit anti-human Exportin-1, monoclonal	Cell Signaling Technology	1:1000 (WB)	Cat# 46249
23	Rabbit anti-human PML, monoclonal	Cell Signaling Technology	1:1000 (WB)	Cat# 33156
24	Rabbit anti-human p-p65 (Ser468), Polyclonal	Cell Signaling Technology	1:1000 (WB)	Cat# 3039
25	Rabbit anti-human Acetyl-p65 (Lys310), monoclonal	Cell Signaling Technology	1:1000 (WB)	Cat# 12629
26	Pacific Blue™ anti-mouse CD4, monoclonal	Biolegend	1:200 (Flow)	Cat# 100427
27	PE anti-mouse CD8b.2, monoclonal	Biolegend	1:200 (Flow)	Cat# 140408
28	FITC anti-mouse CD19, monoclonal	Biolegend	1:200 (Flow)	Cat# 152403
29	APC anti-mouse CD3, monoclonal	Biolegend	1:200 (Flow)	Cat# 100235
30	PE anti-mouse CD25, monoclonal	Biolegend	1:200 (Flow)	Cat# 101903
31	APC anti-mouse/human CD44, monoclonal	Biolegend	1:200 (Flow)	Cat# 103011
32	FITC anti-mouse CD62L, monoclonal	Biolegend	1:200 (Flow)	Cat# 104405
33	Human IFNB1 ELISA	R&D	N/A	Cat# DY814-05
34	Mouse Ifnb1 ELISA	Biolegend	N/A	Cat# 439407
35	Human RANTES ELISA	Biolegend	N/A	Cat# 440807
36	Mouse Rantes ELISA	R&D	N/A	Cat# DY478
37	Mouse Il-17f ELISA	4A BIOTECH	N/A	Cat# CME0089-096
38	TG003	MCE	N/A	Cat# HY-15338
39	MG-132	Selleck	N/A	Cat# S2619
40	Recombinant Human TNF alpha	Novoprotein	N/A	Cat# C008
41	Recombinant Human IL-1 beta	Novoprotein	N/A	Cat# CG93
42	LPS	Sigma	N/A	Cat# L2630
43	Type II Collagenase	Gibco	N/A	Cat# 17101015
44	DNase I	ThermoFisher	N/A	Cat# 18047019
45	Phos-Tag™ Acrylamide	Wako Chemicals	N/A	Cat# 304-93521
46	ADP-Glo™ Kinase Assay	Promega	N/A	Cat# 6930
47	CLK2 Kinase Enzyme System	Promega	N/A	Cat# VA7414

Supplementary Table 2

Supplementary Table 2. The primer sequences and Oligo-DNA sequences

Genes	Primers	Primer sequences	Application
hGAPDH	Forward	5'-GAGTCAACGGATTTGGTCGT-3'	RNA expression
	Reverse	5'-GACAAGCTTCCCGTTCTCAG-3'	RNA expression
mGAPDH	Forward	5'-AGTGTTTCCTCGTCCCGTAG-3'	RNA expression
	Reverse	5'-CCTTGACTGTGCCGTTGAAT-3'	RNA expression
hIFNB1	Forward	5'-TGACTATGGTCCAGGCACAG-3′	RNA expression
	Reverse	5'-TTGTTGAGAACCTCCTGGCT-3′	RNA expression
hRANTES	Forward	5′-GGCAGCCCTCGCTGTCATCC-3′	RNA expression
	Reverse	5′-GCAGCAGGGTGTGGTGTCCG-3′	RNA expression
mIfnb1	Forward	5′-CTGCCTTTGCCATCCAAGAG-3′	RNA expression
	Reverse	5′-TGTCTGCTGGTGGAGTTCAT-3′	RNA expression
mRantes	Forward	5′-ATATGGCTCGGACACCACTC-3′	RNA expression
	Reverse	5′-CACTTGCTGCTGGTGTAGAA-3′	RNA expression
hCLK2	Forward	5′-GTCCGTTCTCGAAGCAGTTA-3′	RNA expression
	Reverse	5′-ACGTTGATCTCAAGTCGAGC-3′	RNA expression
mClk2	Forward	5′-AAGTAGCAGTGACCGGACAA-3′	RNA expression
	Reverse	5′-GTCGTAGTAAGCCTCCCCTC-3′	RNA expression
Clk2 WT	Forward	5'-GCAAGTCGTTCATACCAGCACTC-3'	Mouse genome typing
	Reverse	5'-TGTGGTCCCACAAATGTCTCTC-3'	Mouse genome typing
Clk2 KO	Forward	5'-TTCTAGGAAAGAACGCAGTGGC-3'	Mouse genome typing
	Reverse	5'-GGTGGTATCCGAGACAGGGTTT-3'	Mouse genome typing
sgCLK2	Forward	5'-AGCGACGAAGAAGTCGCTCC-3'	Human gene deletion
	Reverse	5'-GGAGCGACTTCTTCGTCGCT-3'	Human gene deletion
sgClk2-1	Forward	5'-CAGCAATACGATGAGCCTCG-3'	Mouse gene deletion
	Reverse	5'-CGAGGCTCATCGTATTGCTG-3'	Mouse gene deletion
sgClk2-2	Forward	5'-CTGCTTATCCTATCACTCTT-3'	Mouse gene deletion
	Reverse	5'-AAGAGTGATAGGATAAGCAG-3'	Mouse gene deletion
ISD45	Forward	5'-TACAGATCTACTAGTGATCTATGACTGATCTGTACATGATCTACA-3'	DNA ligands
	Reverse	5'-TGTAGATCATGTACAGATCAGTCATAGATCACTAGTAGATCTGTA-3'	DNA ligands
HSV60	Forward	5'-TAAGACACGATGCGATAAAATCTGTTTGTAAAATTTATTAAGGGTACAAATTGCCCTAGC-	DNA ligands
	Reverse	5'-GCTAGGGCAATTTGTACCCTTAATAAATTTTACAAACAGATTTTATCGCATCGTGTCTTA-3	DNA ligands
DNA90	Forward	5'-<br>TACAGATCTACTAGTGATCTATGACTGATCTGTACATGATCTACATACAGATCTACTAGTGA	DNA ligands
	Reverse	5'-<br>TGTAGATCATGTACAGATCAGTCATAGATCACTAGTAGATCTGTATGTAGATCATGTACAG	DNA ligands
HSV120	Forward	5'-<br>AGACGGTATATTTTTGCGTTATCACTGTCCCGGATTGGACACGGTCTTGTGGGATAGGCA	DNA ligands
	Reverse	5'-<br>AAGTCCTCCAAAAAACCCGCCACAAATAAAAAGGGGTTAACCCAATATGCCTTCTGGGCAT	DNA ligands
sgp65^{S180}	Forward	5'-CACCGTGTCAAAGATGGGATGAGAA'	Human gene deletion
	Reverse	5'-AAACTTCTCATCCCATCTTTGACAC'	Human gene deletion
Oligo-DNA-S180A	Forward	5'-<br>T*G*A*CAGTGCGGGACCCATCAGGCAGGCCCCTCCGCCTGCCGCCTGTCCTTGCGCATCCC	Donor
	Reverse	5'-<br>T*G*A*CAGTGCGGGACCCATCAGGCAGGCCCCTCCGCCTGCCGCCTGTCCTTGACCATCCC	Donor
Oligo-DNA-S180D	Forward	5'-<br>T*G*A*CAGTGCGGGACCCATCAGGCAGGCCCCTCCGCCTGCCGCCTGTCCTTGCGCATCCC	Donor
	Reverse	5'-<br>T*G*A*CAGTGCGGGACCCATCAGGCAGGCCCCTCCGCCTGCCGCCTGTCCTTGACCATCCC	Donor
p65 WT	Forward	5'-GCCTGCCGCCTGTCCTTTCT-3'	Human genome typing
p65 180A	Forward	5'-GCCTGCCGCCTGTCCTTGCG-3'	Human genome typing
p65 180D	Forward	5'-GCCTGCCGCCTGTCCTTGAC-3'	Human genome typing
p65 WT	Reverse	5'-CTCTCTCCCCAGCCAACA-3'	Human genome typing
p65 screening	Forward	5'-TATAGAAGAGCAGCGTGGGG-3'	Human genome typing
	Reverse	5'-GCTGGGCAGAGAAGAGTAGA-3'	Human genome typing
CLK2-Left arm	Forward	5'-CCGCTCGAGGATCTTGGGTCCTATCCCTT-3'	Left arm
	Reverse	5'-CCGGAATTCCCGACTGATATCCCGACTGGA-3'	Left arm
CLK2-Right arm	Forward	5'-CTAGCTAGCTATAGCAGTGGGTGTCCAGT-3'	Right arm
	Reverse	5'-AAAAGCGGCCGCTCTGAGCCATGTTTGCAACT-3'	Right arm
sgCLK2 (for Flag-Tag Knock In)	Forward	5'-CACCGGTCGGTGACGATCAGGCCCT-3'	Human gene deletion
	Reverse	5'-AAACAGGGCCTGATCGTCACCGACC-3'	Human gene deletion
hIFNB1 (for ChIP-qPCR)	Forward	5'-TGAAAGGGAGAAGTGAAAGT-3'	ChIP-qPCR
	Reverse	5'-AACACGAACAGTGTCGCCTA-3'	ChIP-qPCR
