# Supplementary material for: CLK2 mediates IκBα-independent early termination of NF-κB activation by inducing cytoplasmic redistribution and degradation
Source: Nat Commun. 2024 May 9;15:3901. doi: 10.1038/s41467-024-48288-z (PMC11082251; doi:10.1038/s41467-024-48288-z)
Supplement: Supplementary file 3 — Reporting Summary [file 41467_2024_48288_MOESM3_ESM.pdf]

nature portfolio

Corresponding author(s): Xiao-Dong Zhang

Last updated by author(s): Apr 11th, 2024

Reporting Summary

Nature Portfolio wishes to improve the reproducibility of the work that we publish. This form provides structure for consistency and transparency in reporting. For further information on Nature Portfolio policies, see our Editorial Policies and the Editorial Policy Checklist.

Statistics

For all statistical analyses, confirm that the following items are present in the figure legend, table legend, main text, or Methods section.

n/a	Confirmed	
☐	☒	The exact sample size (*n*) for each experimental group/condition, given as a discrete number and unit of measurement
☐	☒	A statement on whether measurements were taken from distinct samples or whether the same sample was measured repeatedly
☐	☒	The statistical test(s) used AND whether they are one- or two-sided<br>*Only common tests should be described solely by name; describe more complex techniques in the Methods section.*
☒	☐	A description of all covariates tested
☐	☒	A description of any assumptions or corrections, such as tests of normality and adjustment for multiple comparisons
☐	☒	A full description of the statistical parameters including central tendency (e.g. means) or other basic estimates (e.g. regression coefficient) AND variation (e.g. standard deviation) or associated estimates of uncertainty (e.g. confidence intervals)
☐	☒	For null hypothesis testing, the test statistic (e.g. *F*, *t*, *r*) with confidence intervals, effect sizes, degrees of freedom and *P* value noted<br>*Give P values as exact values whenever suitable.*
☒	☐	For Bayesian analysis, information on the choice of priors and Markov chain Monte Carlo settings
☒	☐	For hierarchical and complex designs, identification of the appropriate level for tests and full reporting of outcomes
☒	☐	Estimates of effect sizes (e.g. Cohen's *d*, Pearson's *r*), indicating how they were calculated

Our web collection on statistics for biologists contains articles on many of the points above.

Software and code

Policy information about availability of computer code

Data collection	Promega GloMax 20/20 for Luciferase; Bio-Rad ChemiDoc XRS+ System for western blot; Monad Real-Time PCR System for real-time PCR; ZEISS LSM 980 for immunofluorescence; BECKMAN CytoFLEX for flow cytometry.
Data analysis	GraphPad Prism 9.0.0 for graphs and statistical analysis; FlowJo X 10.0.7r2 for FACS plots; ImageJ for western blot.

For manuscripts utilizing custom algorithms or software that are central to the research but not yet described in published literature, software must be made available to editors and reviewers. We strongly encourage code deposition in a community repository (e.g. GitHub). See the Nature Portfolio guidelines for submitting code & software for further information.

Data

Policy information about availability of data

All manuscripts must include a data availability statement. This statement should provide the following information, where applicable:

- Accession codes, unique identifiers, or web links for publicly available datasets
- A description of any restrictions on data availability
- For clinical datasets or third party data, please ensure that the statement adheres to our policy

All data generated in this study are provided in the Source Data file. Source data are provided with this paper. The source data underlying Figs. la–h, 2b–e, 3a–b, 3d, 3f–g, 4a–g, 4i, 5a, 5d–e, 5g, 6c–g, 7b–c and 7f–j and Supplementary Figs. 1a, 1c–d, 2a–f, 3a–d, 3g–j, 3l, 4b–d, 5a–d, 5g, 5i, 6a–m, 7a–d. are available in the Source Data file whenever possible.

Research involving human participants, their data, or biological material

Policy information about studies with human participants or human data. See also policy information about sex, gender (identity/presentation), and sexual orientation and race, ethnicity and racism.

Reporting on sex and gender	For sex and gender reporting, we employed equal number of male and female mice for primary cells isolation to reflect the general phenomenon in mice cells. Because female mice have less body weight, and less virus required, we used female mice in virus-induced survival experiments. The remaining male mice were utilized for imiquimod-induced (IMQ) psoriasis-like skin inflammation.
Reporting on race, ethnicity, or other socially relevant groupings	N/A
Population characteristics	N/A
Recruitment	N/A
Ethics oversight	All animal studies were conducted in accordance with the Guidelines of the China Animal Welfare Legislation and approved by the Committee on Ethics in the Care and Use of Laboratory Animals of Wuhan University (permit number: 15060A). All efforts were made to minimize suffering.

Note that full information on the approval of the study protocol must also be provided in the manuscript.

Field-specific reporting

Please select the one below that is the best fit for your research. If you are not sure, read the appropriate sections before making your selection.

☒ Life sciences ☐ Behavioural & social sciences ☐ Ecological, evolutionary & environmental sciences

For a reference copy of the document with all sections, see nature.com/documents/nr-reporting-summary-flat.pdf

Life sciences study design

All studies must disclose on these points even when the disclosure is negative.

Sample size	No statistical method was used to predetermine the sample size. We used generally accepted sample size in accordance to our own previous publication (S.-Z.L. et al., 2019, Nature Communications) with reproducible differences between conditions indicating that the chosen sample size was sufficient. Our sample size is similar to those generally employed in the life sciences field<br>For cell and biochemical experiments, our data usually represent 3 independent biological replicates per condition. For animal experiments, we use 4–6 mice per group.
Data exclusions	No data was excluded from our study.
Replication	The experiments were performed with 2-3 independent replications and each replication is sufficient.
Randomization	For in vitro experiments, cells were randomly allocated into control and experimental groups. For in vivo experiments, age and sex-matched mice were randomized into all experimental groups.
Blinding	the researchers were not blinded during data collection and analysis. The experiments were investigated in specific cell types and with specific treatments and required precise control over the experimental conditions, aspects that are not conditions amenable to blinding. However, the experiments are repeated by independent researchers to validate the results.

Reporting for specific materials, systems and methods

We require information from authors about some types of materials, experimental systems and methods used in many studies. Here, indicate whether each material, system or method listed is relevant to your study. If you are not sure if a list item applies to your research, read the appropriate section before selecting a response.

Materials & experimental systems	
n/a	Involved in the study
☐	☒ Antibodies
☐	☒ Eukaryotic cell lines
☒	☐ Palaeontology and archaeology
☐	☒ Animals and other organisms
☒	☐ Clinical data
☒	☐ Dual use research of concern
☒	☐ Plants

Methods	
n/a	Involved in the study
☒	☐ ChIP-seq
☐	☒ Flow cytometry
☒	☐ MRI-based neuroimaging

Antibodies

Antibodies used

CLK2 (HPA055366) (Sigma, Darmstadt, Germany); CLK2 (A7885), beta-actin (AC026-200), GAPDH (AC033) (Abclonal Technology, Wuhan, China); IRF3 (11904S), p-IRF3 (Ser386) (37829S), p65 (6956S), p-p65 (Ser536) (3033S), IκBα (4812), p-IκBα (2859S), beta-Tubulin (2146S), LaminA/C (4777S), NF-κB1 p105/p50 (13586), CRM1 (46249), PML (33156), p-p65 (Ser468) (3039), Acetyl-p65 (Lys310) (12629) (Cell Signaling, Danvers, MA, USA); Flag (M185-3) (PM020), Myc (M192-3) (562), HA (M180-3) (561) (MBL, Chiba, Japan); p-p65 (Ser180) was purchased form Abclonal Technology; Rabbit IgG HRP Linked Antibody (111-035-003), Mouse IgG HRP Linked Antibody (115-035-003) (Jackson ImmunoResearch, Pennsylvania, USA); Pacific Blue™ anti-mouse CD4 (100427), PE anti-mouse CD8b.2 (140408), FITC anti-mouse CD19 (152403), APC anti-mouse CD3 (100235), PE anti-mouse CD25 (101903), APC anti-mouse/human CD44 (103011), FITC anti-mouse CD62L (104405) (Biolegend, California, USA).

Validation

p-p65 (Ser180) antibody was validated by p65 deficient cell lines in Supplementary Fig. 6k. CLK2 (A7885) (Abclonal Technology, Wuhan, China) was validated in CLK2 deficient cell lines in Supplementary Fig. 3g. All the other antibodies were tested by the manufacturers as below.
CLK2 (HPA055366) (Sigma, Darmstadt, Germany) https://www.sigmaaldrich.cn/CN/zh/product/sigma/hpa055366
beta-actin (AC026-200) (Abclonal Technology, Wuhan, China) https://abclonal.com.cn/catalog/AC026
GAPDH (AC033) (Abclonal Technology, Wuhan, China) https://abclonal.com.cn/catalog/AC033
IRF3 (11904S) (Cell Signaling, Danvers, MA, USA) https://www.cellsignal.cn/products/primary-antibodies/irf-3-d6i4c-xp-174-rabbit-mab/11904
p-IRF3 (Ser386) (37829S) (Cell Signaling, Danvers, MA, USA) https://www.cellsignal.cn/products/primary-antibodies/phospho-irf-3-ser386-e7j8g-xp-174-rabbit-mab/37829
p65 (6956S) (Cell Signaling, Danvers, MA, USA) https://www.cellsignal.cn/products/primary-antibodies/nf-kb-p65-l8f6-mouse-mab/6956
p-p65 (Ser536) (3033S) (Cell Signaling, Danvers, MA, USA) https://www.cellsignal.cn/products/primary-antibodies/phospho-nf-kb-p65-ser536-93h1-rabbit-mab/3033
IκBα (4812) (Cell Signaling, Danvers, MA, USA) https://www.cellsignal.cn/products/primary-antibodies/ikba-44d4-rabbit-mab/4812
p-IκBα (2859S) (Cell Signaling, Danvers, MA, USA) https://www.cellsignal.cn/products/primary-antibodies/phospho-ikba-ser32-14d4-rabbit-mab/2859
beta-Tubulin (2146S) (Cell Signaling, Danvers, MA, USA) https://www.cellsignal.cn/products/primary-antibodies/b-tubulin-antibody/2146
LaminA/C (4777S) (Cell Signaling, Danvers, MA, USA) https://www.cellsignal.cn/products/primary-antibodies/lamin-a-c-4c11-mouse-mab/4777
NF-κB1 p105/p50 (13586) (Cell Signaling, Danvers, MA, USA) https://www.cellsignal.cn/products/primary-antibodies/nf-kb1-p105-p50-d4p4d-rabbit-mab/13586
CRM1 (46249) (Cell Signaling, Danvers, MA, USA) https://www.cellsignal.cn/products/primary-antibodies/exportin-1-crm1-d6v7n-rabbit-mab/46249
PML (33156) (Cell Signaling, Danvers, MA, USA) https://www.cellsignal.cn/products/primary-antibodies/pml-e5r8t-rabbit-mab/33156
p-p65 (Ser468) (3039) (Cell Signaling, Danvers, MA, USA) https://www.cellsignal.cn/products/primary-antibodies/phospho-nf-kb-p65-ser468-antibody/3039
Acetyl-p65 (Lys310) (12629) (Cell Signaling, Danvers, MA, USA) https://www.cellsignal.cn/products/primary-antibodies/acetyl-nf-kb-p65-lys310-d2s3j-rabbit-mab/12629
Flag (M185-3) (MBL, Chiba, Japan) https://www.mblbio.com/bio/g/dtl/A/index.html?pcd=M185-3L
Flag (PM020) (MBL, Chiba, Japan) https://www.mblbio.com/bio/g/dtl/A/?pcd=PM020
Myc (M192-3) (MBL, Chiba, Japan) https://www.mblbio.com/bio/g/dtl/A/?pcd=M192-3
Myc (562) (MBL, Chiba, Japan) https://www.mblbio.com/bio/g/dtl/A/?pcd=562
HA (M180-3) (MBL, Chiba, Japan) https://www.mblbio.com/bio/g/dtl/A/?pcd=M180-3
HA (561) (MBL, Chiba, Japan) https://www.mblbio.com/bio/g/dtl/A/?pcd=561
Rabbit IgG HRP Linked Antibody (111-035-003) (Jackson ImmunoResearch, Pennsylvania, USA) https://www.jacksonimmuno.com/catalog/products/111-035-003
Mouse IgG HRP Linked Antibody (115-035-003) (Jackson ImmunoResearch, Pennsylvania, USA) https://www.jacksonimmuno.com/catalog/products/115-035-003
Pacific Blue™ anti-mouse CD4 (100427) (Biolegend, California, USA) https://www.biolegend.com/en-gb/products/pacific-blue-anti-mouse-cd4-antibody-3316
PE anti-mouse CD8b.2 (140408) (Biolegend, California, USA) https://www.biolegend.com/en-gb/products/pe-anti-mouse-cd8b-2-antibody-6871
FITC anti-mouse CD19 (152403) (Biolegend, California, USA) https://www.biolegend.com/en-gb/products/fitc-anti-mouse-cd19-antibody-13615
APC anti-mouse CD3 (100235) (Biolegend, California, USA) https://www.biolegend.com/en-gb/products/apc-anti-mouse-cd3-antibody-8055
PE anti-mouse CD25 (101903) (Biolegend, California, USA) https://www.biolegend.com/en-gb/products/pe-anti-mouse-cd25-

antibody-129
APC anti-mouse/human CD44 (103011) (Biolegend, California, USA) https://www.biolegend.com/en-gb/products/apc-anti-mouse-human-cd44-antibody-312
FITC anti-mouse CD62L (104405) (Biolegend, California, USA) https://www.biolegend.com/en-gb/products/fitc-anti-mouse-cd62l-antibody-384

Eukaryotic cell lines

Policy information about cell lines and Sex and Gender in Research

Cell line source(s)	HEK293T, HeLa, THP-1 and Vero cell lines were come from ATCC (Zhenghe Wang and Hongbing Shu' lab previously used); The p65-/- HEK293T cell line was a gift from Bo Zhong (Wuhan University, Hubei, China); CLK2-/- HEK293T cells and p65S180A/D knock-in cell lines were generated in our lab; BMDMs and BMDCs were isolated from 6-week-old C57BL/6J Clk2 knockout mice (two male mice and two female mice); MLFs, Lungs were isolated from 10-week-old mice (two male mice and two female mice).
Authentication	Common used HEK293T, HeLa, THP-1 and Vero cell lines which were previously used in Zhenghe Wang and Hongbing Shu' lab were identified by commpany. Murine cell lines were derived by ourself and authenticated by morphology.
Mycoplasma contamination	Mycoplasma contamination was detected for common used cell lines, 293T and THP1. And there is no contamination of Mycoplasma
Commonly misidentified lines (See ICLAC register)	There is no commonly misidentified cell lines were used in the study.

Animals and other research organisms

Policy information about studies involving animals; ARRIVE guidelines recommended for reporting animal research, and Sex and Gender in Research

Laboratory animals	The male or female C57BL/6 mice at 6-10 weeks were used for the experiments and housed under a 12:12-hour light/dark cycle at a controlled temperature.
Wild animals	There is no wild animals were used in the study.
Reporting on sex	There was no sex bias in the animals used in this study.
Field-collected samples	There is no field collected samples were used in the study.
Ethics oversight	All animal studies were conducted in accordance with the Guidelines of the China Animal Welfare Legislation and approved by the Committee on Ethics in the Care and Use of Laboratory Animals of Wuhan University (permit number: 15060A). All efforts were made to minimize suffering.

Note that full information on the approval of the study protocol must also be provided in the manuscript.

Plants

Seed stocks	N/A
Novel plant genotypes	N/A
Authentication	N/A

Flow Cytometry

Plots

Confirm that:

☒ The axis labels state the marker and fluorochrome used (e.g. CD4-FITC).

☒ The axis scales are clearly visible. Include numbers along axes only for bottom left plot of group (a 'group' is an analysis of identical markers).

☒ All plots are contour plots with outliers or pseudocolor plots.

☒ A numerical value for number of cells or percentage (with statistics) is provided.

Methodology

Sample preparation	After 16 hours of GFP-VSV infection, the indicated cells were harvested and resuspended in 0.5 mL phosphate-buffered saline (PBS); Single cell suspensions were incubated with fluorochrome-conjugated antibodies against surface markers in PBS containing 1.5% FBS for 20 min at 4 C and then washed. Cells were then fixed for 30 min at 4 C using Biolegend Cytofix/ Cytoperm and washed twice followed by flow cytometry analysis.
Instrument	The samples were analyzed by flow cytometry (Cytoflex, Beckman Coulter, Fullerton, CA, USA)
Software	The flow cytometry data were collected by cytoexpert (Beckman Coulter, Fullerton, CA, USA) and analyzed using FlowJo software (TreeStar, Ashland, OR, USA)
Cell population abundance	around 10000-20000 cells were collected
Gating strategy	For GFP-VSV infection,According to the intensity of GFP; Forward versus side scatter (FSC vs SSC) gating was used to identify cells of interest and exclude debris and dead cells. Also, A forward scatter height (FSC-W/A) vs. forward scatter area (FSC-H) density plot was used to exclude doublets.

☒ Tick this box to confirm that a figure exemplifying the gating strategy is provided in the Supplementary Information.
